# Higher serum chloride concentrations are associated with acute kidney injury in unselected critically ill patients

**DOI:** 10.1186/1471-2369-14-235

**Published:** 2013-10-28

**Authors:** Zhongheng Zhang, Xiao Xu, Haozhe Fan, Danyu Li, Hongsheng Deng

**Affiliations:** 1 Department of Critical Care Medicine, Jinhua Municipal Central Hospital, Jinhua Hospital of Zhejiang University, 351#, Mingyue Road, 321000 Jinhua, Zhejiang Province, China

**Keywords:** Acute kidney injury, Chloride, Intensive care unit, Critically ill

## Abstract

**Background:**

Chloride administration has been found to be harmful to the kidney in critically ill patients. However the association between plasma chloride concentration and renal function has never been investigated.

**Methods:**

This was a retrospective study conducted in a tertiary 24-bed intensive care unit from September 2010 to November 2012. Data on serum chloride for each patient during their ICU stay were abstracted from electronic database. Cl_0_ referred to the initial chloride on ICU entry, Cl_max_, Cl_min_ and Cl_mean_ referred to the maximum, minimum and mean chloride values before the onset of AKI, respectively. AKI was defined according to the conventional AKIN criteria. Univariate and multivariable analysis were performed to examine the association of chloride and AKI development.

**Results:**

A total of 1221 patients were included into analysis during study period. Three hundred and fifty-seven patients (29.2%) developed AKI. Cl_max_ was significantly higher in AKI than in non-AKI group (111.8 ± 8.1 vs 107.9 ±5.4 mmol/l; p < 0.001); Cl_0_ was not significantly different between AKI and non-AKI patients; Cl_mean_ was significantly higher in AKI than non-AKI (104.3 ±5.8 vs 103.4 ± 4.5; p = 0.0047) patients. Cl_max_ remained to be associated with AKI in multivariable analysis (OR: 1.10, 95% CI: 1.08-1.13).

**Conclusion:**

Chloride overload as represented by Cl_mean_ and Cl_max_ is significantly associated with the development of AKI.

## Background

Acute kidney injury is a common complication of varieties of critical illness and acts as a major contribution to the high mortality rate in intensive care unit (ICU) [1-3]. A large body of evidence suggests that even a mild increase in serum creatinine will have significant negative impact on all-cause mortality [4,5]. Thus, great effort has been made to elucidate the underlying mechanisms of the development of AKI, based on which preventive or therapeutic strategies can be developed to control this devastating complication. Although there are numerous strategies for the prevention and treatment of AKI including optimization of hemodynamic status, use of vasodilators (e.g. dopamine and fenoldopam), early initiation of continuous renal replacement therapy and use of natriuretic peptides, most of them failed to show a beneficial effect [6-10].

Critically ill patients receive large amount of intravenous fluid administration during their ICU stay. Many commercially available crystalloid fluids are rich in chloride; for example, the most widely used saline 0.9% has 40% higher chloride than human plasma. It is intuitive that the administration of such non-physiological fluid will impair the balance of internal homeostasis. Some animal studies suggest that administration of chloride-liberal fluid induces renal vasoconstriction and a decline in glomerular filtration rate [11,12]. More recently, Yunos NM and colleagues [13] conducted a sequential period pilot study in unselected critically ill patients and found that the incidence of AKI can be reduced by 50% by using chloride-restrictive fluids. However, these studies are preliminary and the association between chloride and kidney function remains to be elucidated. The present study aimed to investigate the association of plasma chloride and the development of AKI in critically ill patients. We hypothesized that plasma chloride was associated with the development of AKI during ICU stay.

## Methods

### Study population and settings

This was a retrospective cohort study conducted in a tertiary 24-bed mixed ICU from September 2010 through November 2012. The hospital was a large academic medical center with 2000 beds. All patients admitted to the ICU during study period were potentially eligible for the present analysis. All clinical data were abstracted from Haitai e-chart (Haitai Medical information systems Co.,LTD). Exclusion criteria were 1) patients with preexisting renal impairment as represented by a serum creatinine > 135 μmol/l in the past 30 days before this ICU admission; 2) patients with incomplete medical information that prohibit analysis; 3) patients transferred to other hospitals before recovery; and such patients were labeled as “automatically discharged” in e-chart; 4) patients had no chloride measured during their ICU stay; 5) patients younger than 18 years old. The study was approved by the ethic committee of Jinhua municipal central hospital and informed consent was waived due to retrospective nature of the study.

Physiological scores for the severity of illness such as acute physiology and chronic health evaluation (APACHE) score and sequential organ failure assessment (SOFA) score were not routinely obtained for each patients; thus, we used the well validated Charlson’s comorbidity index for risk adjustment [14,15]. Chloride was measured by automated chemistry analyzer (Sysmex, Sysmex Asia Pacific Pte Ltd and Sysmex Corporation of Japan). The reference range of chloride was 98 to 106 mmol/l. To explore the temporal association of chloride values and AKI development, only chloride values before the onset of AKI were obtained from database. For patients without development of AKI, all available chloride values during ICU stay were obtained. Cl_0_ referred to the initial chloride on ICU entry, Cl_max_, Cl_min_ and Cl_mean_ referred to the maximum, minimum and mean chloride values before the onset of AKI, respectively. Because most critically ill patients had more than one diagnosis, we used the primary diagnosis (the one in the first sheet of medical chart) for the classification of patients, as well as for subgroup analysis.

The primary outcome was the development of AKI as defined by an increase in serum creatinine by >50% from baseline [16]. If baseline creatinine was not available, it was estimated by assuming that glomerular filtration rate (GFR) in that given patient was 75 ml/min per 1.73 m^2^, and serum creatinine can be estimated by using the modified 4-variable Modification of Diet in Renal Disease (MDRD) formula [17,18]. AKI patients were further classified into AKIN-1,-2 and-3 according to acute kidney injury network (AKIN) report [19]. Secondary outcomes included hospital and ICU length of stay (LOS), hospital mortality, use of continuous renal replacement therapy (CRRT). If a patient had more than one episode of ICU stay, ICU LOS was the sum of each.

### Statistical analysis

Demographic and clinical data were compared between AKI and non-AKI patients. Parametric data were expressed using mean ± standard deviation (SD) and compared using t test; non-parametric data were expressed as median and interquartile range (IQR) and compared using Wilcoxon rank-sum test. To determine the dose–response relationship between chloride and AKI, chloride values were compared among groups without AKI, with AKIN-1,-2 and-3 by using one-way analysis of variance (ANOVA). Multiple comparisons were made by using Bonferroni method. Variables with p < 0.1 in bivariate analysis were incorporated into Logistic regression model for risk adjustment. The goodness-of-fit of the regression model was tested using Homser-Lemeshow χ^2^ test. Sensitivity analysis was performed by excluding patients with missing baseline creatinine. All analyses were performed using the software StataSE 11.2 (College Station, Texas 77845 USA). Conventional two-tailed p < 0.05 was considered to be statistically significant.

## Results

A total of 1583 patients were identified during study period. Three hundred and sixty-two of them were excluded because 352 had preexisting renal dysfunction and 10 had incomplete medical information. As a result, a total of 1221 patients were eligible for current analysis (Table 1). Baseline serum creatinine levels were missing in 203 patients. There were 357 patients (29.2%) developed AKI during their ICU stay. As compared with non-AKI, patients with AKI were older (62.0 ± 19.8 vs 58.8 ± 19.5 years; p = 0.011), had higher Charlson comorbidity index (1[0-2] vs 0[0-2]; p = 0.0095), more likely to use mechanical ventilation (62.8% vs 43.3%, p < 0.001), and had higher hospital mortality (27.2% vs 18.2%;p < 0.001), longer hospital LOS (29 [20,41] vs 22 [15,33],p < 0.001), and longer ICU LOS (4 [3,9] vs 3 [2,6]; p < 0.001). Patients in coronary care setting were less likely to develop AKI (8% vs 14.4%; p = 0.002); and there was no difference in the incidence of AKI in medical, surgical and post-cardiac surgery patients. AKI patients had more fluid balance (1245 ± 782 vs 984 ± 780 ml; p < 0.001) and greater amount of saline 0.9% infusion (1052 ± 768 vs 954 ± 650 ml; p = 0.023) than non-AKI patients. Cl_0_ was not significantly different between AKI and non-AKI patients (104.1 ± 7.3 vs 103.4 ± 5.4 mmol/l; p = 0.07). Cl_max_ was significantly higher in AKI than in non-AKI patients (111.8 ± 8.1 vs 107.9 ±5.4 mmol/l; p < 0.001), and Cl_min_ was significantly lower in AKI than in non-AKI (97.2 ± 6.1 vs 98.3 ± 5.6 mmol/l; p = 0.002). Cl_mean_ was significantly higher in AKI than in non-AKI patients (104.3 ±5.8 vs 103.4 ± 4.5 mmol/l; p = 0.0047).

**Table 1:** Baseline characteristics of included patients

**Variables**	**Total (n = 1221)**	**AKI (n = 357)**	**Non-AKI (n = 864)**	**P value**
Age (years)	59.7 ± 19.6	62.0 ± 19.8	58.8 ± 19.5	0.011
Sex (male, %)	788 (64.5%)	233 (65.3%)	556 (64.2%)	0.117
Charlson index (median, IQR)	0 (0-2)	1 (0-2)	0 (0-2)	0.0095
Primary Classification (n, %)				<0.001
Medical	340 (27.8)	111 (31.1)	229 (26.5)	0.104
Coronary	152 (12.4)	28 (8.0)	124 (14.4)	0.002
Post-cardiac surgery	101 (8.0)	22 (6.0)	79 (9.0)	0.085
Surgical	628 (51.4)	196 (54.9)	432 (50.0)	0.119
Fluid balance (ml)†	1061 ± 789	1245 ± 782	984 ± 780	<0.001
Saline 0.9% administration (ml)†	983 ± 678	1052 ± 768	954 ± 650	0.023
Use of diuretics (n, %)	408 (33.42)	115 (32.21)	293 (33.91)	0.567
Use of MV (n; %)	598 (49.0)	224 (62.8)	374 (43.3)	<0.001
Survivors (n; %)	967 (79.2)	260 (72.8)	707 (81.8)	<0.001
Hospital LOS (days; median, IQR)^‡^	24 (16,35)	29 (20,41)	22 (15,33)	<0.001
ICU LOS (days; median, IQR) ^‡^	3 (2,7)	4 (3,9)	3 (2,6)	<0.001
Bicarbonate (mmol/l)	23.9 ± 3.0	24.0 ± 3.2	23.9 ± 2.9	0.46
Cl_0_ (mmol/l)	103.6 ±6.0	104.1 ± 7.3	103.4 ± 5.4	0.07
Cl_mean_ (mmol/l)	103.7 ±4.9	104.3 ±5.8	103.4 ± 4.5	0.0047
Cl_min_ (mmol/l)	98.0 ± 5.8	97.2 ± 6.1	98.3 ± 5.6	0.002
Cl_max_ (mmol/l)	109.1 ± 6.6	111.8 ± 8.1	107.9 ±5.4	<0.001

^‡^Analysis was restricted to survivors; † data obtained on the day of the onset of acute kidney injury or on the day of the highest serum creatinine level.

*Abbreviations*: *AKI* acute kidney injury, *IQR* interquartile range, *MV* mechanical ventilation, *Cl*_*0*_ chloride measured on ICU entry, *Cl*_*mean*_ mean chloride during ICU stay, *Cl*_*min*_ minimum chloride during ICU stay, *Cl*_*max*_ maximum chloride during ICU stay.

In multiple-comparison test, a dose–response relationship was identified between Cl_max_ and AKIN stages (Table 2). AKIN-1 had significantly higher Cl_max_ than non-AKI (111.0 ± 7.4 vs 107.9 ± 5.4 mmol/l; p < 0.001), and AKIN-3 had significantly higher Cl_max_ than AKIN-2 (115.9 ± 11.0 vs 111.7 ± 7.3; p < 0.05). Table 3 shows the adjustment of odds ratio with multiple regression analysis. After adjustment, Cl_max_ and Cl_mean_ remained to be significantly associated the development of AKI with odds ratio of 1.10 (95% CI: 1.08-1.13, p < 0.001) and 1.04 (95% CI: 1.02-1.07, p = 0.002), respectively. In patients with AKI, a total of 45 patients required renal replacement therapy. However, none of Cl_min_, Cl_max_ or Cl_mean_ was significantly different between AKI patients with and without CRRT (data not shown).

**Table 2:** Dose–response relationship between chloride values and the severity of AKI

	**Non-AKI**	**AKIN-1**	**AKIN-2**	**AKIN-3**
Cl_0_ (mmol/l)	103.4 ± 5.4	103.4 ± 6.6	104.2 ± 6.6	106.7 ± 10.8^¶^
Cl_max_ (mmol/l)	107.9 ± 5.4	111.0 ± 7.4^¶^	111.7 ± 7.3^¶^	115.9 ± 11.0 ^¶^
Cl_min_ (mmol/l)	98.3 ± 5.6	97.3 ± 6.0	97.0 ± 5.2	96.7 ± 8.4
Cl_mean_ (mmol/l)	103.4 ± 4.5	103.8 ± 5.5	104.2 ± 5.1	106.7 ± 7.8^¶^

Note: Differences among the four groups were tested using one-way analysis of variance (ANOVA), and multiple comparisons were performed using Bonferroni method.

^¶^Indicates the chloride value in the present AKI stage is significantly higher than that in the milder stage in the left cell. For example, Cl_max_ in AKIN-1 was significantly higher than that in non-AKI (108.9 vs 107.8, p < 0.001).

**Table 3:** Logistic regression model to test independent variables associated with AKI

**Variables**	**Odds ratio**	**95% confidence interval**	**P**
Cl_0_	1.02	0.998-1.05	0.06
Cl_max_	1.10	1.08-1.13	<0.001
Cl_min_	0.96	0.94-0.98	0.001
Cl_mean_	1.04	1.02-1.07	0.002

*Abbreviations*: *MV* mechanical ventilation.

Note: the Logistic regression model was fitted by incorporating all potentially confounding factors including bicarbonate level, use of diuretics, age, sex, comorbidity score and primary diagnosis.

**Figure 1:**
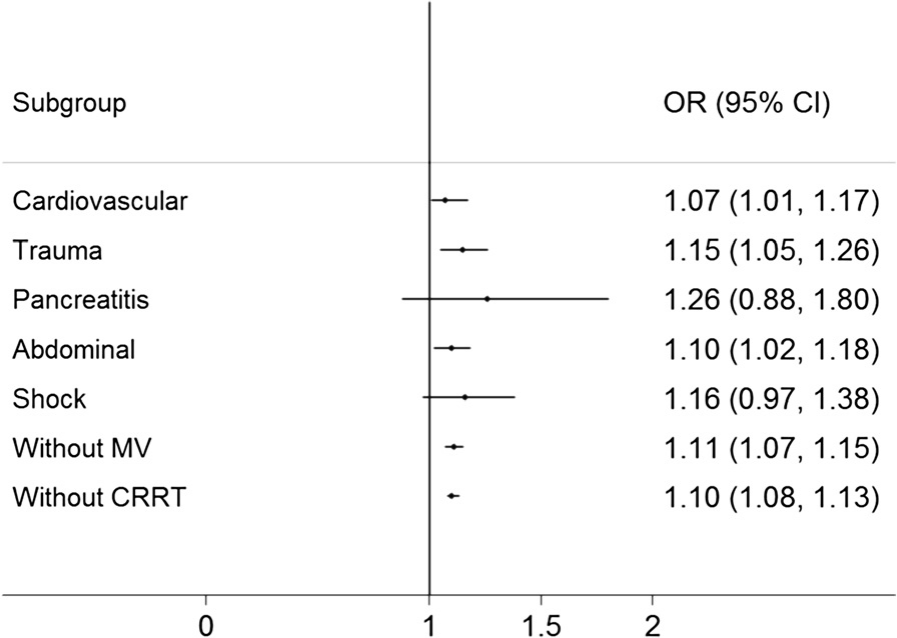
**Subgroup analysis restricting to patients with cardiovascular diseases, trauma, pancreatitis, abdominal problem, shock, without MV and without CRRT.** OR, odds ratio; MV, mechanical ventilation; CRRT, continuous renal replacement therapy.

Subgroup analysis was performed by restricting to patients with cardiovascular diseases, trauma, pancreatitis, abdominal problem, shock, without MV and without CRRT. The results were generally consistent with the overall analysis (Figure 1). Sensitivity analysis by excluding patients with missing baseline creatinine was performed, and the results remained unchanged (data not shown).

## Discussion

Our study for the first time demonstrated that higher Cl_max_ and Cl_mean_ were associated with subsequent development of AKI, while higher Cl_min_ was protective against the development of AKI. Furthermore, Cl_max_ was positively correlated to the severity of AKI as staged by AKIN criteria.

Our result supports the notion that excessive chloride intake has negative impact on renal function. In animal study, Wilcox CS showed that chloride can induce vasoconstriction and such effect is specific for the renal vessels [11]. Other negative effects of chloride on kidney include the reduction in cortical perfusion and the increase in systemic inflammation. In a clinical trial, Wu BU and colleagues [20] compared Ringer’s solution and saline 0.9% on their effect on inflammatory response. The result showed that patients who received saline 0.9% appeared to have higher C-reactive protein (CRP) and systemic inflammatory response syndrome (SIRS) levels. Since SIRS is a well known risk factor for AKI, it is not surprising that elevated inflammatory level will pose the kidney at increased risk of injury [21-23]. In another study, Chowdhury AH and colleagues [24] found that administration of saline 0.9% ([Cl] 154 mmol/L) significantly reduced renal cortical tissue perfusion and mean renal artery flow velocity as compared to the infusion of Plasma-Lyte 148 ([Cl] 98 mmol/L). Alternative explanation for the linkage between higher chloride and AKI may be that these patients are more hypotensive and require more saline. Another finding in our study is that Cl_min_ is protective against the development of AKI, which can be explained by the fact that the optimal serum chloride is within the normal range, and hypochloremia has negative impact on renal function. However, there is no compelling evidence demonstrating the negative impact of hypochloremia, and this mandates further investigations. In a landmark study, Yunos NM and coworkers [13] only reported that chloride-restrictive arm received significantly less amount of chloride infusion during study period (496 vs 694 mmol per patient), but did not report serum chloride levels in both arms. This limits the interpretation of the finding with respect to whether use of chloride-restrictive fluid will benefit overall population or just patients with hyperchloremia. According to our findings, we speculate that chloride-restrictive strategy may not benefit patients with already existing hypochloremia. Thus, it is of great interest to conduct a randomized controlled trial by stratifying patients according to their baseline serum chloride levels.

Several other studies show neutral effect of chloride-restrictive strategy in protecting renal function [25-29]. However, these studies are performed in patients with contrast-induced nephropathy (CIN). It is probable that the protective effect of chloride-restrictive fluid is not universal and can only be found in critically ill patients. In CIN setting, the total amount of chloride infusion is limited even in chloride-libel arm. In contrast, during fluid resuscitation in ICU setting the amount of infused fluid is much larger than that in CIN setting, allowing the beneficial effect of chloride restriction large enough to be detected.

In our study, we failed to identify an association between chloride and the use of renal replacement therapy (RRT). This is in contrast to the study by Yunos NM and coworkers in which chloride-restrictive strategy is associated with reduced need for RRT. However, because we excluded patients with preexisting renal impairment on ICU entry, the number of patients requiring RRT is extremely low in our study (4%). Such a small sample size significantly limited the statistical power of our study. Secondly, indications for the initiation of RRT vary considerably across different areas and institutions, and thus the comparison between our findings and results from other institutions should be cautious.

Many biomarkers in critical care medicine are found to be predictive of clinical outcomes. For the prediction of mortality, CRP, B-type natriuretic peptide and red cell distribution width are found to be promising; [30,31] and for the prediction of AKI, cystatin C and NGAL are thought to be promising [32,33]. Such early identification of adverse outcome may inform clinicians to adopt preventive strategies to reduce the risk of adverse outcome. The clinical implication of our finding is that while initial chloride value is not predictive of the AKI development, the handling of chloride during treatment is of vital importance. For a patient with hyperchloremia on ICU entry, the use of chloride-restrictive strategy will effectively reduce the risk of AKI. Conversely, if excessive saline 0.9% is infused to a patient with normal serum chloride level, he or she will be put on high risk of AKI development. However, due to the observational nature of our study; the result can only be served as hypothesis-generating. Further well designed controlled trials may help to solve this uncertainty [34].

There are several limitations in our study that should be mentioned in interpretation of the results. First, the measurement of serum chloride was not scheduled and the obtained Cl_max_ and Cl_min_ might not reflect the true values. Second, disease severity score such as SOFA or APACHE ǁ was not incorporated into analysis, which significantly limited the reliability of our result. However, we used Charlson’s comorbidity index, which is accessible from administrative database, for risk stratification. Although not as good as disease severity scores involving physiologic parameters, Charlson’s index has been validated for use in critical care setting [14,15]. Third, although we have tried our best to control bias and confounders by using multivariate model, many other known or unknown factors may still exist. Thus, the conclusion of the study is at best hypothesis-generating, and further experimental studies are mandatory to confirm current findings.

## Conclusion

In aggregate, the study shows that higher Cl_mean_ and Cl_max_ are associated with the development of AKI, indicating that chloride overload during ICU treatment may increase the risk of AKI. Furthermore, higher Cl_min_ is found to be protective against AKI, indicating that restricting chloride infusion is no longer beneficial in patients with hypochloremia.

## Competing interests

The authors declare that they have no competing interests.

## Authors’ contributions

ZZ conceived of the study, participated in the statistical analysis and drafted the manuscript. XX carried out the data abstraction. HF participated in the data abstraction and statistical analysis. DL participated in the design of the study and performed the statistical. HD participated in study design and coordination. All authors read and approved the final manuscript.

## Pre-publication history

The pre-publication history for this paper can be accessed here:

http://www.biomedcentral.com/1471-2369/14/235/prepub
